# Consequences of a short time exposure to a sublethal dose of Flupyradifurone (Sivanto) pesticide early in life on survival and immunity in the honeybee (*Apis mellifera*)

**DOI:** 10.1038/s41598-019-56224-1

**Published:** 2019-12-24

**Authors:** Yahya Al Naggar, Boris Baer

**Affiliations:** 1 Center for Integrative Bee Research (CIBER), Department of Entomology, University of California Riverside, Riverside, CA 92507 USA; 2 Zoology Department, Faculty of Science, Tanta University31527, Tanta, Egypt; 3 General Zoology, Institute for Biology, Martin Luther University Halle-Wittenberg, Hoher weg 8, 06120 Halle (Saale), Germany

**Keywords:** Molecular ecology, Entomology

## Abstract

Dramatic losses of pollinating insects have become of global concern, as they threaten not only key ecosystem services but also human food production. Recent research provided evidence that interactions between ecological stressors are drivers of declining pollinator health and responsible for observed population collapses. We used the honeybee *Apis mellifera* and conducted a series of experiments to test for long-term effects of a single short exposure to the agricultural pesticide flupyradifurone to a second environmental stressor later in life. To do this, we exposed individuals during their larval development or early adulthood to sublethal dosages of flupyradifurone (0.025 μg for larvae and 0.645 μg for imagos), either pure or as part of an agricultural formulation (Sivanto). We afterwards exposed bees to a second ecological stressor infecting individuals with 10,000 spores of the fungal gut parasite *Nosema ceranae*. We found that pesticide exposures significantly reduced survival of bees and altered the expression of several immune and detoxification genes. The ability of bees to respond to these latter effects differed significantly between colonies, offering opportunities to breed bees with elevated levels of pesticide tolerance in the future. We conclude that short episodes of sublethal pesticide exposures during development are sufficient to trigger effects later in life and could therefore contribute to the widespread declines in bee health.

## Introduction

Insect pollination is of central importance for human food production and ecosystem stability^1,2^ but has become of global concern given recent reports of substantial declines in several native^3,4^ and managed bee populations^2,5,6^. Despite a substantial amount of research that has been conducted over recent years to identify and study the different ecological factors that contribute towards continuous declines in pollinator populations, we still lack a complete understanding about the observed losses of these key insects. A number of factors have been identified as contributing factors such as pesticide exposure^7,8^, parasite infections^9,10^, habitat loss^4^, climate change^11^ or inferior pollinator management^12^. However, no single factor has so far been able to fully explain the observed declines in pollinator populations.

Recent research provided empirical evidence that combinations of sublethal ecological stressors can result in additive or synergistic effects that drive declines in pollinator health^7,13–15^. However, a detailed understanding of how individual environmental stressors impact pollinating insects solely or in combination still remains poorly understood^16^. Effects of co-exposure to multiple ecological stressors have been studied in the honey bee *Apis mellifera*, where they can increase mortality^15,17–20^, reduce the survival of queens^21^ and drone fertility^15,22^ or increase parasite susceptibility^19,23^. Although these studies provide independent empirical support for the presence of additive or synergistic effects of environmental stressors, their future management will require more detailed insights into the sophisticated dynamics between the individual stressors, such as for example their impact at different life history stages, possible long term effects over time and beyond their direct exposure or their short or long term impacts on the physiology, i.e. on the genomic and proteomic level.

Pesticides are well documented drivers of declining bee health^7,8,24,25^. Here, we used a relatively new systemic pesticide flupyradifurone (Sivanto) for our experiments, which reversibly binds to insect nicotinic acetylcholine receptors (nAChR) and is therefore similar to other insecticides used widely in agricultural and urban environments such as neonicotinoids and sulfoximines^26^. So far, only few studies investigated the impact of flupyradifurone (FLU) on bees and to our knowledge, there are no previous studies available that quantified potential interaction effects between this pesticide and other ecological stressors. Exposure to high, non-field-realistic FLU dosages of 1.2 μg per bee were found to impair their taste, cognition^27^ and motor sensory abilities^28^. Chronic exposure to FLU at field-realistic concentrations impaired olfactory learning in *Apis cerana* larva (0.033 μg / day) and workers (0.066 μg /day)^29^. No adverse effects were found on the colony level following exposure to maximum label rate^30^. This latter study, funded by the manufacturer of the pesticide, was criticized however, because nectar and pollen samples from control sites were also contaminated with the pesticide^31^.

Effects of FLU on honeybees have also been compared to those of other pesticides such as the fungicide SBI (sterol biosynthesis inhibitor) propiconazole^32^. Co exposure to both chemicals at field realistic dosages resulted in synergistic effects and over proportionally reduced survival and behavioral activity. What remains to be studied though are effects of sub lethal exposure during different developmental stages and specific tests whether known pesticide induced damages are reversible or have long-term impacts. Such information could be crucial for future beekeeping and pesticide application practices.

Honeybees harbor a large number of different parasites^33–35^ that differ substantially in virulence and also contribute towards the documented losses of honeybee stock. The microsporidian *Nosema ceranae*^36,37^ is a globally widespread fungal disease of honeybees, that infects and multiplies in the midgut epithelia of adult honeybees. In the absence of additional environmental stressors, chronic *Nosema* infections do not seem to be drivers of colony losses^38^ as they do not result substantial increases in mortality^22,34^ but cause various subtle effects such as for example an earlier commencement of foraging^39,40^. However, severe infections have been linked to reduced lifespans of foragers and increased colony mortality^39,41,42^.

Here, we conducted a series of experiments where we exposed honeybee workers for a very brief period of time during their larval development and early adulthood to sub lethal, field relevant concentrations of FLU. We afterwards quantified long-term effects on survival and immunocompetence in the absence of the pesticide. To expose bees to a second ecological stressor, we infected them with *N. ceranae* and compared phenotypic and genotypic responses of non-exposed larvae or adults with those exposed to the parasite, the pesticide or both stressors. This allowed us to quantify whether short episodes of pesticide exposures early in life have long lasting effects and impact key life history traits such as immunocompetence well beyond exposure times.

## Results

### Effects of pesticide and parasite exposure on honeybee larvae

#### Survival

**Figure 1:**
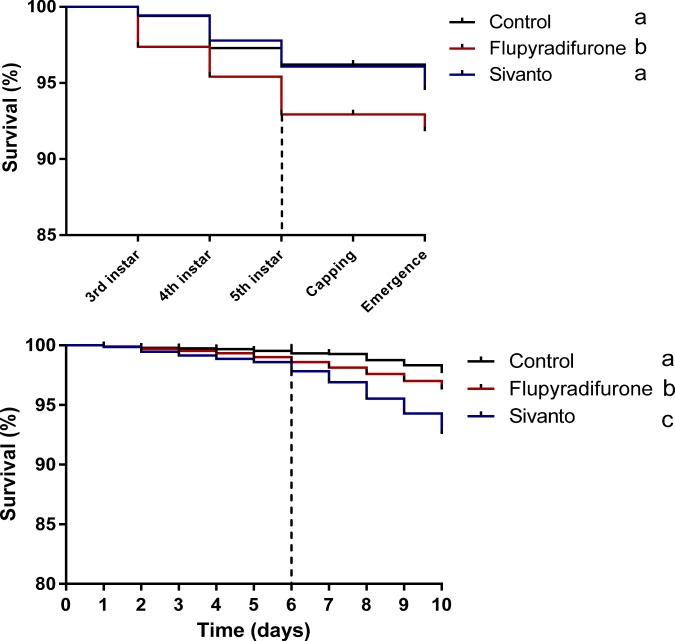
Kaplan–Meier plot showing the survival of honeybee workers between the different pesticide treatments. (**a**) *Honeybee larvae were fed with 0.025* *µg of the pesticide per larvae compared to non-exposed control individuals during their early larval development*. (**b**) *Honeybee workers were fed each with 0.65* *µg of the pesticide either pure or as part of an agricultural formulation (Sivanto)* during their early adult stage. Letters indicate statistical differences between treatments after Bonferroni correction (log-rank (Mantel cox) paired test, *p* < 0.01). Dashed line indicates the end of the pesticide exposure time.

We found that exposure to FLU significantly reduced larval survival prior to cell capping compared to larvae fed with sugar water (Log-rank (Mantel-Cox) Test: (χ^2^ = 19.17, *p* = 0.016; after Bonferroni correction **(**Fig. 1a)). Overall larval mortality was low (7 ± 2% (mean ± sem) however, resulting in sufficient numbers of individuals surviving and becoming available for further experiments.

#### Parasite infection

**Figure 2:**
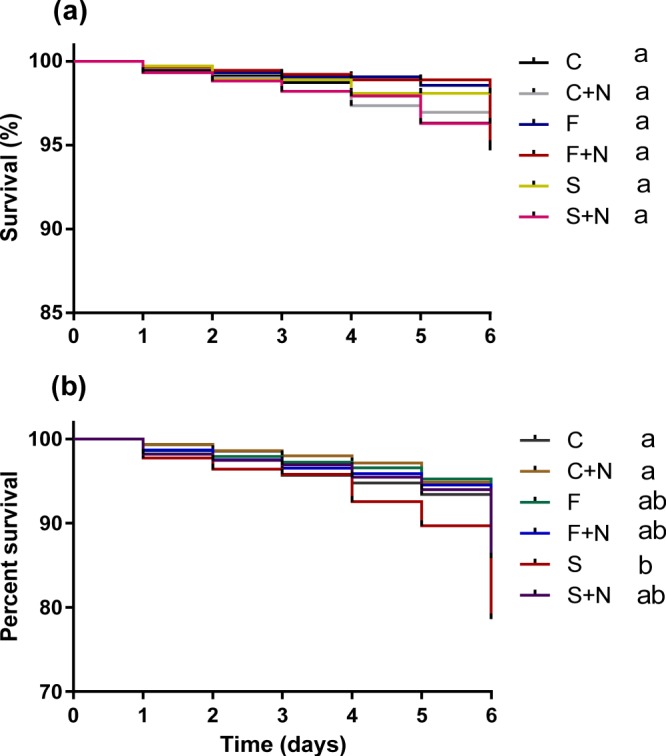
Kaplan–Meier plot showing survival of honeybee workers between the different parasite and pesticide treatments. (**a**) Bee larvae were exposed to 0.025 μg pesticide and infected with 10,000 *N. ceranae* spore per bee four days after eclosion. (**b**) Newly eclosed bees were fed with sublethal doses of pesticides (0.645 μg for imagos) and consequently infected with *N*. *ceranae* (10,000 spore per bee) when they were 12 days old. Different letters denote significant statistical differences between treatments (log-rank (Mantel cox) paired test, *p* < 0.005, after Bonferroni correction). C; control, C + N; control + *N.ceranae*, F; FLU, F + N; FLU + *N.ceranae*, S; Sivanto, S + N; Sivanto + *N.ceranae*.

**Figure 3:**
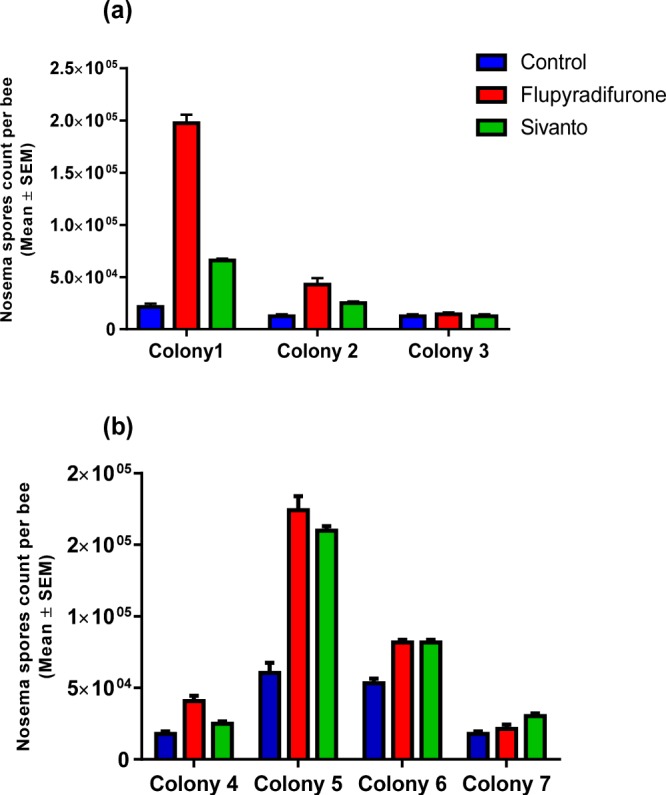
Mean (±SEM). *Nosema ceranae* infection intensities between treatments and colonies. (**a**) Individuals that were exposed to pesticides (0.025 μg for larvae) during larval development. (**b**) Bees fed with pesticides (0.645 μg for imagos) during their early adult life.

We used 900 surviving workers (300 per treatment) to test whether pesticide exposure during larval development affected disease tolerance during adulthood. We found no significant difference in the survival between *Nosema*-infected and non-infected bees, independently of larval feeding treatment (Log-rank (Mantel-Cox) Test: χ^2^ = 5.18, *p* = 0.39 after Bonferroni correction, Fig. 2a) or the amount of food consumed (GLM, Wald Chi square 0.355: *P* = 0.64, see Supplementary Information Fig. S1 and Table S1 for full statistical details**)**. However, when we compared infection intensities in 66 workers, or 9 ± 2 (mean ± sem) individuals per treatment, we found a significant colony x pesticide interaction (GLM, Wald χ2 = 161.23, p < 0.001 Fig. 3a, see Table 1 for statistical details), indicating that the effect of pesticide exposure on *Nosema* infection intensity differed between colonies. Pesticide exposure resulted in higher *Nosema* infections intensities in 2 out of the 3 colonies studied, (Fig. 3a). As expected, no *Nosema* infections were found in non-infected control bees.

**Table 1:** Results from statistical GLM analyses testing effects of Flupyradifurone (Sivanto) exposure on *Nosema ceranae* infection intensity.

Source	Type III		
	df	Wald Chi-Square	Sig.
**Bees exposed to pesticides during larval treatment**			
Intercept	1	1226.85	<0.001
Colony	2	390.45	<0.001
Treatment	2	261.16	<0.001
Colony * Treatment	4	161.23	<0.001
**Bees exposed to pesticides during early adult stage**			
Intercept	1	2795.26	<0.001
Colony	3	437.14	<0.001
Treatment	2	121.31	<0.001
Colony * Treatment	6	41.71	<0.001

Exposure to the pesticide resulted in higher *N. ceranae* intensities compared to non-exposed bees, but a significant colony x treatment interaction term indicated that the bee’s ability of respond to the parasite infections differed between colonies. (see Fig. 3).

#### Gene expression

**Figure 4:**
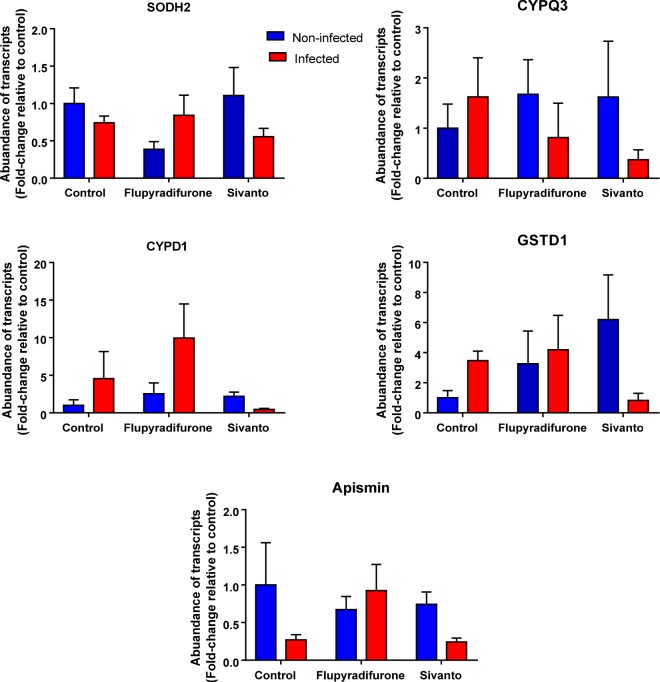
Fold-change in abundances of transcripts of some detoxification and immunity related genes whose expressions were significantly impacted in individuals co-exposed to pesticides (0.025 μg for larvae) during larval stages and later to *N. ceranae* (10,000 spore per bee) during early adult life compared to control. For statistical details see Table 2, bars depict (Mean ± SEM).

When we compared the expression of 5 detoxification and 6 immune genes between infected and non-infected bees that were fed with FLU, Sivanto or a control not containing the pesticide, we found significant parasite x pesticide interaction terms for the 4 detoxification genes SODH2, CYPQ3, CYPD1 and GSTD1 and for the immune gene Apismin (*p* < 0.05: see Table 2 for complete statistical details) indicating that the expression of these genes in response to *Nosema* infections depended on whether these individuals were exposed to the pesticide or not during their larval development. Gene expression was always lower in infected bees co-exposed to both the pesticide and the parasite compared to non-infected or non-exposed control bees **(**Fig. 4).

**Table 2:** Results from GLM analyses testing for effects of Flupyradifurone (Sivanto) and/or *N. ceranae*-infection on the expression of 5 different detoxification and 6 different immunity genes in honeybees.

Source	df	Type III (*P*-value)										
		Pesticides exposure during larval development										
		Detoxification genes					Immunity genes					
		SODH2	CYPS14	CYPQ3	CYPD1	GSTD1	Chitinase	Hymeno	Defensin	Apismin	Lys-1	PGRPS2
(Intercept)	1	<0.01	<0.01	<0.01	<0.01	<0.01	<0.01	<0.01	<0.01	<0.01	<0.01	<0.01
Nosema	1	0.01	<0.01	0.09	0.05	0.44	0.09	0.90	0.73	0.01	0.07	0.82
Flupyradifurone	2	0.65	0.27	0.68	0.03	0.23	0.25	0.36	0.07	0.02	1.00	0.61
Colony	2	0.36	<0.01	<0.01	0.35	<0.01	0.23	0.24	0.08	<0.01	0.06	0.08
Nosema* Flupyradifurone	2	0.02	0.63	0.02	0.02	<0.01	0.43	0.23	0.86	<0.01	0.12	0.09
**Pesticides exposure during early adult stage**												
(Intercept)	1	<0.01	<0.01	<0.01	<0.01	<0.01	<0.01	0.05	<0.01	<0.01	<0.01	<0.01
Nosema	1	0.30	0.90	0.96	0.07	0.63	0.01	0.30	0.02	0.11	0.60	0.47
Flupyradifurone	2	0.49	0.05	0.17	0.09	0.21	0.13	0.46	0.06	0.01	0.02	0.37
Colony	3	0.19	0.04	0.01	0.14	0.39	0.08	0.64	0.37	<0.01	0.01	0.01
Nosema* Flupyradifurone	2	0.41	0.35	0.08	0.16	0.21	0.99	0.37	0.20	0.05	0.86	0.06

Significant interaction terms in several genes indicated that expression changes found depended on whether or not individuals were previously exposed to the pesticide. (See Fig. 4 for more details).

### Effects of pesticide and parasite exposure on adult honeybee workers

#### Survival

We found that the exposure to low levels of pesticide either pure or as part of an agricultural formulation (Sivanto) during early adult life significantly reduced survival of bees as compared to that of non-exposed control bees (Log-rank (Mantel-Cox) Test: (FLU, χ^2^ = 9. 73, *p* = 0.016 after Bonferroni correction; Sivanto: χ^2^ = 48. 39, *p* = 0.016). Survival of workers exposed to the formulation Sivanto was furthermore significantly lower compared to those exposed to flupyradifurone solely (Log-Rank Test) (χ^2^ = 15.48, *p* = 0.016) (Fig. 1b). We found no significant differences on average daily sugar water consumption between the three treatment groups (GLM: *P* = 0.27) (See Supplementary Information Fig. S2a, Table S2 for statistical details).

#### Parasite infections

We used 1100 surviving workers (275 ± 5 workers per colony) to test for any effects of pesticide exposure on *Nosema* susceptibility. We found that the survival of bees exposed to Sivanto was significantly lower compared to control bees ((Log-Rank Test), χ^2^ = 9.67, *p* = 0.001) and infected bees ((Log-Rank Test), χ^2^ = 14.38, *p* = 0.0001 < 0.005 after Bonferroni correction) **(**Fig. 2b**)**. When we compared the amount of food consumed, we found a significant interaction term (pesticides x parasite) (GLM, Wald Chi square 6.319: *P* = 0.04) (See Supplementary Information Fig. S2b, Table S3 for statistical details), indicating that food consumption of *Nosema*-infected bees depended on whether or not individuals were previously exposed to the pesticide.

When we compared *Nosema* intensity in a total of 72 workers (12 ± 2 mean ± sem per colony) that were infected and/or pesticide exposed, we found a significant colony x pesticide interaction term (GLM, Wald χ^2^ = 41.71, *p* < 0.001 Fig. 3b, see Table 1 for statistical details), indicating that the effects of pesticide exposure differed between colonies. In 2 out of the 4 colonies, *Nosema* infections were higher in bees exposed to the pesticide compared to controls, and higher infection intensities in individuals exposed to FLU compared to Sivanto (Fig. 3b). As expected, we did not find any *Nosema* infections in bees of the non-infected control groups, irrespectively of pesticide treatment.

#### Gene expression

**Figure 5:**
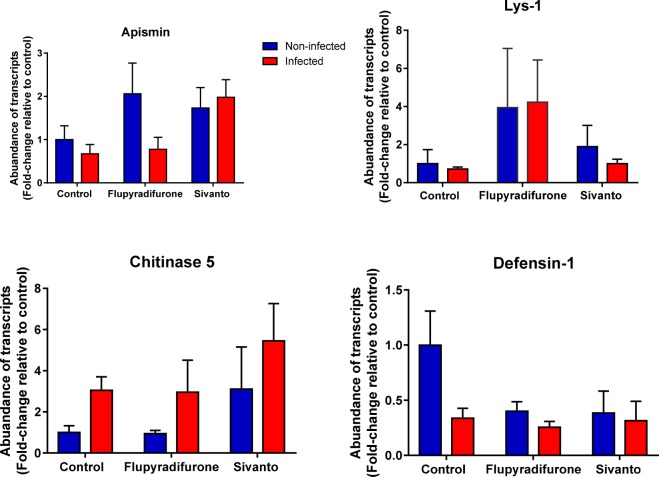
Differences in gene transcription of several immune genes in 18-day old individuals exposed to either pesticides (0.645 μg for imagos) and / or *N*. *ceranae* (10,000 spores per bee) compared to non-exposed/infected control bees. For statistical details see Table 2, bars depict (Mean ± SEM).

When we compared gene expression of 6 immune and 5 detoxication genes between the 3 pesticide and 2 parasite treatments, no significant pesticide x parasite interaction terms were found for any of the genes investigated (Table 2) compared to control bees. We found a significantly higher expression of chitinase in honeybees infected with *N. ceranae* (*P* = 0.01) (Fig. 5) and a significantly lower expression of the immune peptide defensin (*P* = 0.02) compared to the non-infected control bees. Apismin and Lys-1 expression were significantly higher in pesticide exposed bees compared to control bees (*P* < 0.02) irrespectively of whether they were infected or not (Fig. 5 and Table 2 for complete statistical details).

## Discussion

We found that sublethal exposures to FLU indeed impacted bees well beyond their exposure time and in the paragraphs below, we first discuss the toxicity levels of the pesticide found in our experiments, followed by a discussion about the effects of pesticide exposure and parasite infection on immunocompetence and the expression of key immune- and detoxification genes.

We found a significant reduction in survival of pesticide exposed larvae and adult bees although absolute mortalities observed were relatively low. This could be different under natural conditions, if developing larvae are continuously fed with contaminated food, for example if pesticide containing pollen or nectar becomes stored and used later^43^. Our experimental setup aimed to mimic natural rearing and development conditions as much as possible and therefore followed earlier developed protocols investigating similar questions^29^, but future work could also conduct follow up experiments *in vitro*. The latter should be feasible, given that the necessary methods have already been developed and successfully used for previous research^44–46^. More work is also required to quantify effects of observed pesticide induced mortalities on the colony level and whether they can impact their performance and reproductive success.

Our findings are in line with earlier reports that exposure to other pesticides, herbicides and fungicides during larval development negatively impact the life history of bees later in life^29,47,48^. Consequently, quantifying effects of pesticide exposure on bee life history traits requires experimental setups that conduct long term monitoring as effects may only become visible after a time lag and later in the life cycle^49^. Although, there is mounting evidence that formulations of pesticides result in higher levels of toxicity than the individual active substances^50,51^, we found no consistent differences between FLU or its formulated product Sivanto on bees. Our findings are in line with earlier work reporting inconsistent effects of neonicotinoid-based formulations or their active ingredients in water fleas *(Daphnia magna)*^52^.

When we infected bees with a parasite of low virulence to quantify immunocompetence, we found significant differences between colonies (genotypes). Infection intensities were higher in pesticide exposed bees in some colonies, but others seemed to be able to mitigate these effects because their infection intensities were not statistically different from those of the control treatments. Such variation between colonies was also reported for differential transcription of immune/detoxifying genes in honeybee larvae in response to exposure to the herbicide glyphosate^48^. Although our sample size on the colony level was low, our findings should be followed up with future research because genetic variation in immune responsiveness could be used to unravel the underlaying physiological mechanisms to understand the genetic basis of possible pesticide tolerance, for example whether certain colonies differ in the expression of detoxification or immune genes compared to susceptible colonies. Such knowledge could also be used to breed bees in the future that are better able to cope with sublethal pesticide exposures in the future.

As expected, our parasite treatment did not result in any significant increases in bee mortality. Overall, pesticide exposed bees had higher infection intensities, which has also been reported in several previous studies^19,23^. The consequences of accelerated parasite infections could be substantial on the colony level, if pesticide exposed bees spread infections faster to other colony members inside their hive or to other colonies via the shared use of flowers. The latter would mean that pesticide exposed bees and/or their colonies could act as important disease vectors on the population level and to other pollinators, and such intraspecies disease transferees have indeed become of concern^53^. We obtained a single measure of infection intensities to compare effects of pesticide exposure on disease susceptibility, but further work should quantify infections over time, which could reveal important insights into the subtle effects of the pesticide on host-immunocompetence and the potential consequences of pesticide exposure on disease transmission risk.

Differences in gene expression were surprisingly different between larvae and early adults, indicating that the timing of a pesticide exposure is important and impacts individuals differentially. Overall, our data imply that effects of pesticide exposure are more pronounced in larvae compared to young adults^20,23^. This is remarkable given that there was a time span of four weeks between the pesticide exposure in larvae and the measures of gene expression in adults, which is almost spanning the total life expectancy of honeybee workers^54^. This indicates that bees that are exposed early in life to a pesticide are substantially more susceptible to environmental stress once they start foraging^55,56^, which could have important ramifications on the colony level if this results in a reduction in total foraging time or efficiency. Future work is therefore required to quantify the effects of pesticide exposure on foraging efficiency and length, for example using already established methods of tracking bees with radio frequency tags in the field^57,58^.

## Methods

### Honeybee breeding

All experiments were carried out between August and October 2018 using honeybees (*Apis mellifera*) that we kept in an apiary at the University of California Riverside (USA). Prior to any research activities, we initially standardized the size of 7 experimental colonies by providing them with 7 frames of worker brood, a single empty frame for queens to lay eggs and 8 frames of wax foundation. We allowed colonies to recover for 7 days and confirmed at the start of experimental work that colonies were in generally good health, as indicated by the presence of a single, egg laying queen, worker brood, honey and pollen storage and the absence of any pathological signs of diseases.

### Pesticide inocula

We purchased flupyradifurone as the pure chemical (FLU ≥ 98% purity, Sigma Aldrich, USA) as well as part of a formulation used for seed treatments during agricultural applications (Sivanto 200SL_,_ Bayer CropScience, USA). Because flupyradifurone is easily soluble in water (3,200 mg/ L at 20 °C), we therefore dissolved FLU or Sivanto in dd H2O to obtain stock solutions with a concentration of 1 μg/μl. We prepared new working solutions at the start of each experiment or replicate by diluting subsamples of stock solutions with sugar water containing 500 g/l of sucrose to a final concertation of 0.0043 µg / µl FLU. We kept the working solutions on ice at 4 °C and wrapped them in aluminum foil.

Residual contaminations of FLU in nectar and pollen have been quantified for several plants such as oilseed rape (4.3 ppm in nectar and 21 ppm in pollen), cotton nectar (22 ppm), apple pollen (39 ppm) and blueberry pollen (68 ppm)^59^. Tosi & Nieh^32^ used these values to calculate maximal, field-realistic FLU oral exposure levels for bees. They concluded that individual workers could become exposed up to 5.5 µg FLU per day through nectar and up to 2.4 µg FLU through pollen if they forage on treated oilseed rape and up to 1.56 µg FLU per foraging flight in cotton fields. Here we exposed individuals during their larval development or early adulthood to significantly lower dosages of flupyradifurone, using 0.025 μg for larvae and 0.645 μg for imagos, either pure or as part of an agricultural formulation (Sivanto). Our pesticide exposure levels were therefore an order of magnitude lower than estimated field relevant dosages per single foraging day^32^ and also substantially lower than published LD_50_ (1.2 µg/bee) values^60^.

### *Nosema ceranae* spore collection and infection

Sampling of *Nosema* spores was performed using a protocol developed earlier^61^. In brief, we collected 100 foraging worker bees at the entrances of five hives with known *N. ceranae* infections, that were different to those we used for the breeding of experimental honeybees. We dissected their midguts and pooled them into a single Eppendorf tube containing 1 mL of deionized water and a single 3 mm tungsten bead (Qiagen, USA). We homogenized the sample using a mixer mill (Retsch MM301) at 25 Hz for 30 s, layered 500 µl of the homogenate onto 1.5 ml of 100% Percoll (Sigma-Aldrich) in a 2 mL Eppendorf tube and centrifuged the sample for 90 min at 20,000 × g and 4 °C. We discarded the supernatant and resuspended the pellet in 1 mL double distilled water, briefly mixed the sample before centrifugation for 10 minutes at 28,000 × g and 4 °C. This process was repeated for a total of 4 times before we resuspended the final pellet in DDI water. We quantified spore concentration using a Neubauer haemocytometer and diluted the spore sample with 50% (w/v) sucrose solution to obtain a final concentration of 5,000 spores/µl. To inoculate bees, we used a micrpipette and hand fed bees with either 2 µl of 50% (w/v) sucrose solution containing 10,000 spores or 2 µl sucrose solution as control. Earlier work confirmed that such infection intensities reliably trigger infection but do not result in significant increases in mortality^62^. As *Nosema apis* seems absent in bee populations in Southern California (McFrederick, personal communication), we assumed that our inoculation samples consisted of *N. ceranae* only. Even if *N. apis* would have been present in our *Nosema* sample, this would not impact our results and conclusions, because we used a single collection of spores for all experiments and all inoculated bees were therefore exposed to the same spore cocktail.

### Pesticide exposure during larval development

To quantify consequences of FLU and Sivanto exposure on honeybees during their larval development, we confined the queens of three different colonies onto a caged worker comb for up to 30 hours to allow them to lay eggs. We released the queen and kept the egg containing frames in their maternal colonies for 3 days to allow eggs to hatch. We then recollected frames and randomly assigned three areas containing 120–130 larvae each to one of three treatments (control, FLU, Sivanto) before returning them back to their colonies for another 2 days to allow larval development to the 3^rd^ instar. A total of 1125 larvae, or 375 ± 5 (mean ± sem) individuals per colony consequently became available for experiments. In a next step we used a 10 µl micropipette and mixed 2 µl of inoculate with the larval food present at the bottom of brood cells. Inocula contained either sucrose solution with 0.0043 µg /µl of FLU (either pure or as part of Sivanto) or sucrose solution only as a control. Because we repeated this feeding regime over three consecutive days, we provided larvae of the two pesticide treatments with a total FLU dosage of 0.025 µg per individual, which is equivalent to 2.1% of the LD_50_^60^. After each feeding treatment, we followed a protocol published earlier^29^ and kept frames in an incubator for 2 hours at 34 °C at 50–60% humidity to ensure that larvae consumed the inocula before returning them to their maternal hives. We checked frames on a daily basis and recorded the number of surviving larvae, the number of sealed cells as well as the number of emerging bees. For the latter, we removed brood after 19 days from colonies, separated the brood areas according to treatment and transferred them to small wooden cages that we kept in an incubator in the lab at 34 °C and 50–60% humidity.

We collected all workers four days after the first individuals had emerged and randomly assigned them to one of two treatments, feeding half of them with sugar water containing 10,000 *N. ceranae* spores and the other half with sugar solution solely as a control. To do this, we starved bees for 2 hours and then hand fed individual bees with 2 µl inoculum using a 10 µl micropipette. We afterwards kept bees in plastic cages^46^ and provided them with sugar water *ad libitum*, separated by infection treatment (infected versus control) and pesticide treatment (FLU, Sivanto and control). Worker mortality and food consumption were quantified daily, and all surviving bees were collected after 6 days to quantify *Nosema* infection intensity and the expression of several immunity and detoxification genes as described below.

### Pesticide exposure during early adult life

We also quantified the consequences of FLU and Sivanto exposure in adult bees. To do this we used four colonies that were different to those we used for the larval experiments. For each colony, we transferred a single frame with sealed worker brood to an incubator in the lab kept at 34 °C. We collected 360 newly emerging bees per colony, transferred them to plastic cages and provided them with sugar water ad libitum, or sugar water containing FLU or Sivanto (0.0043 µg/µl). We consequently ended up with a total of 1440 workers that became available for further experimentation. We removed all treatment feeds after 6 days and provided bees afterwards with sugar water *ad libitum* for another 4 days. For a total of 10 days we quantified sugar water consumption and worker survival daily. All surviving workers were randomly allocated to one of two treatments and inoculated with either sucrose solution containing 10,000 *N. ceranae* spores or sucrose solution only as a control. We kept workers for an additional 6 days in cages separated by the 6 treatments and provided them with sugar water *ad libitum*. We quantified worker mortality and food consumption daily and used surviving bees to quantify *Nosema* intensity and gene expression as described below.

### *Nosema* intensity quantification

We quantified the number of *Nosema* spores in the midgut of bees using three randomly selected workers per treatment and colony that had survived until the end of the experiment. To do this we, dissected their midguts into a single Eppendorf tube containing 1 mL of deionized water and a single 3 mm tungsten bead (Qiagen, USA). We homogenized the sample using a mixer mill (Retsch MM301) at 25 Hz for 30 s, layered 500 µl of the homogenate onto 1.5 ml of 100% Percoll (Sigma-Aldrich) in a 2 mL Eppendorf tube and centrifuged the sample for 90 min at 20,000 × g and 4 °C. We discarded the supernatant and resuspended the pellet in 1 mL double distilled water for 10 minutes at 28,000 × g and 4 °C. This process was repeated 4 times before we resuspended the final pellet in 50 µl DDI water. We used a 10 μl subsample to quantify spores’ numbers using a Neubauer chamber and a Leica light microscope at 400× magnification. Total spore number per sample was calculated by multiplying spores counts by 50.

### Gene expression

We used real-time quantitative PCR (RT-qPCR) to measure for any effects of pesticide and/or *N. ceranae* exposure on the expression of several key immune and detoxification genes that have been used for comparable studies in the past^18,63^. We used six genes with well documented involvement in insect immune responses such as defensin1, apismin, hymenopteacin, PGRPS2, Lys-1 which are all part of the Toll/Antimicrobial peptide or the Imd pathway^64,65^. We also selected chitinase because of its well demonstrated antifungal activity^66^ and because it is upregulated in response to *Nosema* infections in honeybees^63,67^. We selected an additional 5 genes with well documented detoxifying activities using CYP305D1, CYP6AS14, CYP9Q3 as representatives of the cytochrome P450 pathway and GSTD & SODH2 as representatives of antioxidant- enzyme families that are known to target pesticides and secondary metabolites as part of a detoxification response in honeybees^63,68^.

The primers for all genes were taken from^18,63^ and purchased from Integrated DNA Technologies (IDT, USA) (Supplementary information Table S4). To quantify gene expression, we isolated total RNA by pooling the guts of 6 surviving bees from both the larval the adult exposure experiment and for each of the 6 treatments separately. We used a RNeasy Lipid Tissue Mini Kit (Qiagen, CA, USA) following the manufacturer’s instructions to extract RNA from our samples which we quantified using a Nanodrop ND-1000 spectrometer (Nanodrop Technologies, Wilmington, DE). We synthesized a first strand of cDNA using 0.5 µg of RNA extract per sample and a Quantitect cDNA Synthesis Kit (Qiagen) and used a LightCycler 96 Real-Time PCR System (Biorad CFX 96, USA) to conduct RT-qPCR using Quantitect SYBR Green Reagent (Qiagen). We used two technical replicates of 20 µL per sample and all samples were transferred to single well on a 96-well PCR plate (Bio-Rad, USA).

Samples were denatured at 95 °C for 15 min prior to RT-qPCR. A total of 40 thermal cycles were run using the manufacturers specifications: 15 s at 94 °C, 30 s at 55 °C and 30 s at 72 °C. The RT-qPCR cycle was followed by a dissociation step to validate that only a single product was amplified in each reaction. For each target gene, abundance of transcripts was quantified according to the Mean Normalized Expression (MNE) method of^69^, and actin-related protein-1 (AMActin) was used as a reference gene. We also determined primer efficiency using standard curves of serial dilutions of cDNA. We confirmed acceptable reaction conditions for all genes because the coefficient of determination (*R*^2^) was >0.99 and efficiencies ranged between 85–110% **(**Table S4**)**, as provided by the PCR machine.

### Data analyses

All statistical analyses were performed using SPSS 24 for Windows. To compare survival between treatments we used Kaplan–Meier log-rank paired tests. To test for treatment effects on food consumption and gene expression, we used generalized linear models (GLMs) with normal distributions and identity link function. Pesticide exposure, pathogen infection and colony were used as independent factors. To test for any significant effects of co-exposure to pesticides and parasitism, we inspected the pathogen x pesticide interaction terms and therefore kept them in all models, independently of whether they were statistically significant or not. To compare *Nosema* infection intensities between the three pesticide treatments, we used GLMs using pesticide exposure and colony as independent factors.

## Supplementary information

Consequences of a short time exposure to a sublethal dose of Flupyradifurone (Sivanto) pesticide early in life on survival and immunity in the honeybee (Apis mellifera)

## Data Availability

The datasets generated during and/or analyzed during the current study are available from the corresponding author on reasonable request.
