# Supplementary material for: Consequences of a short time exposure to a sublethal dose of Flupyradifurone (Sivanto) pesticide early in life on survival and immunity in the honeybee (Apis mellifera)
Source: Sci Rep. 2019 Dec 24;9:19753. doi: 10.1038/s41598-019-56224-1 (PMC6930273; doi:10.1038/s41598-019-56224-1)
Supplement: Supplementary file 1 — Consequences of a short time exposure to a sublethal dose of Flupyradifurone (Sivanto) pesticide early in life on survival and immunity in the honeybee (Apis mellifera) [file 41598_2019_56224_MOESM1_ESM.docx]

**Consequences of a short time exposure to a sublethal dose of Flupyradifurone (Sivanto) pesticide early in life on survival and immunity in the honeybee (*Apis mellifera*)**

Yahya Al Naggar^1,2,3^, Boris Baer^1^

^1^Center for Integrative Bee Research (CIBER), Department of Entomology, University of California Riverside, Riverside, CA 92507, USA

^2^Zoology Department, Faculty of Science, Tanta University31527, Tanta, Egypt.

^3^General Zoology, Institute for Biology, Martin Luther University Halle-Wittenberg, Hoher weg 8, 06120 Halle (Saale), Germany,

* Correspondence:

[boris.bar@ucr.edu](mailto:boris.bar@ucr.edu);

[yehia.elnagar@science.tanta.edu.eg](mailto:yehia.elnagar@science.tanta.edu.eg)

**Table S1**: Results of GLM analysis testing for effects of Flupyradifurone (Sivanto) pesticide exposure and *Nosema ceranae* infection on food consumption of honeybee workers exposed to pesticides during their early larval development.

|  | | | |
| --- | --- | --- | --- |
|  | | | |
| Source | Type III | | |
|  | Wald Chi-Square | df | Sig. |
| (Intercept) | 604.678 | 1 | < 0.001 |
| Nosema | 1.024 | 1 | .599 |
| Pesticides | .083 | 2 | .773 |
| Colony | 3.725 | 2 | .155 |
| Nosema * Pesticides | .355 | 2 | .837 |

**Table S2**: Results of a GLM analysis testing for effects of Flupyradifurone (Sivanto) pesticide exposure on food consumption of honeybee workers exposed to pesticides during their early adult life.

| Source | | Type III Sum of Squares | df | F | *P*-value |
| --- | --- | --- | --- | --- | --- |
| Intercept |  | 8194.891 | 1 | 140.484 | .001 |
| pesticides |  | 66.539 | 2 | 1.623 | .273 |
| colony |  | 175.000 | 3 | 2.846 | .128 |

**Table S3**: Results of a GLM analysis testing for effects of Flupyradifurone (Sivanto) pesticide exposure and *Nosema ceranae* infection on food consumption of honeybee workers exposed to pesticides during their early adult life.

| Source | Type III | | |
| --- | --- | --- | --- |
|  | Wald Chi-Square | df | Sig. |
| (Intercept) | 1905.849 | 1 | < 0.001 |
| Nosema | .022 | 1 | .882 |
| Pesticides | 2.617 | 2 | .270 |
| Colony | 10.197 | 3 | .017 |
| Nosema * Pesticides | 6.319 | 2 | .**042** |

**Table S4.** Primer sequences and their efficiencies for quantification of abundances of transcripts in honeybees (*Apis mellifera*).

| **Locus** | **Category** | **F. Primer** | | | **R. Primer** | | | **Efficiency** |
| --- | --- | --- | --- | --- | --- | --- | --- | --- |
| **Cytochrome p450 (CYP305D1)** | **Detoxification** | **TCGATCTTTTTCTCGCTGGT** |  |  | **TTGCTTTGTCCTCCATGTTG** |  | **1.85** | |
| **Cytochrome p450 (CYP6AS14)** | **Detoxification** | **TGAAACTCATGACCGAGACG** |  |  | **AAAATTTGGGCCGCTAATAAA** |  | **1.91** | |
| **Cytochrome p450 (CYP9Q3)** | **Detoxification** | **GTTCCGGGAAAATGACTAC** |  |  | **GGTCAAAATGGTGGTGAC** |  | **1.91** | |
| **Glutathione-S-transferases (GSTD1)** | **Detoxification** | **GCCGCTTCAAAAGAAGTACG** |  |  | **GTGGCGAAAACAAGGATGAT** |  | **2.04** | |
| **Superoxide dismutase (SODH2)** | **Detoxification** | **CAGTGCATGGTAGCCTGAGA** |  |  | **ACAGTGCTCCTTCAGCCAAT** |  | **1.95** | |
| **Peptidoglycan-recognition protein (PGRPS2)** | **Immunity** | **TAATTCATCATTCGGCGACA** |  |  | **TGTTTGTCCCATCCTCTTCC** |  | **1.97** | |
| **Saccharopine dehydrogenase [NAD(+), L-lysine-forming] (Lys-1)** | **Immunity** | **GAACACACGGTTGGTCACTG** |  |  | **ATTTCCAACCATCGTTTTCG** |  | **1.98** | |
| **Defensin-1** | **Immunity** | **TGCGCTGCTAACTGTCTCAG** |  |  | **AATGGCACTTAACCGAAACG** |  | **2.02** | |
| **Hymenopt** | **Immunity** | **CTCTTCTGTGCCGTTGCATA** |  |  | **CGTCTCCTGTCATTCCATT** |  | **2.08** | |
| **Apisimin** | **Immunity** | **TGAGCAAAATCGTTGCTGTC** |  |  | **AACGACATCCACGTTCGATT** |  | **1.99** | |
| **Chitinase 5** | **Immunity** | **TATCGAAAGGACGTCGGCAG** |  |  | **TCACGCCGATGAACGAGTAG** |  | **1.96** | |
| **Actin related protein 1 (β-actin)** | **House keeping** | **ATGCCAACACTGTCCTTTCTGG** |  |  | **GACCCACCAATCCATACGGA** |  | **1.98** | |

**Fig. S1.** Effects of exposure to Flupyradifurone (Sivanto) pesticide and *Nosema ceranae* infection on food consumption (the volume of consumed food per day per bee) (Mean ± SEM) of honeybee workers exposed to pesticides during their early larval treatment.

**Fig.S2.** Effects of exposure to : A) Flupyradifurone (Sivant) pesticide on food consumption (the volume of consumed food per day per bee) (Mean ± SEM) of honeybee workers exposed to pesticides during their early adult life., B) Flupyradifurone (Sivanto) pesticide exposure and *Nosema ceranae* infection on food consumption of honeybee workers exposed to pesticides during their early adult.
